# Descriptive Epidemiology of Early-Onset Gastrointestinal Cancers in Iran, 2014-2018

**DOI:** 10.34172/mejdd.2024.365

**Published:** 2024-01-31

**Authors:** Mohammad Sadra Gholami Chahkand, Fatemeh Esmaeilpour Moallem, Fatemeh Ghasemi-Kebria, Reza Malekzadeh, Gholamreza Roshandel, Mohammad Taher

**Affiliations:** ^1^Golestan Research Center of Gastroenterology and Hepatology, Golestan University of Medical Sciences, Gorgan, Iran; ^2^Digestive Oncology Research Center, Digestive Diseases Research Institute, Shariati Hospital, Tehran University of Medical Sciences, Tehran, Iran; ^3^Division of Gastroenterology and Hepatology, Imam Khomeini Hospital Complex, Tehran University of Medical Sciences, Tehran, Iran; ^4^Liver Transplantation Research Center, Tehran University of Medical Sciences, Tehran, Iran

**Keywords:** Early-onset, Esophageal cancer, Gastric cancer, Colorectal cancer, Iran

## Abstract

**Background:**

We aim to present incidence rates and geographical distribution of most common early-onset gastrointestinal cancers (EOGICs), including early-onset esophageal cancer (EOEC), gastric cancer (EOGC) and colorectal cancer (EOCRC) in Iran, 2014-2018.

**Methods:**

Data on new cases of EOEC, EOGC and EOCRC were obtained from publicly available annual reports of the Iranian National Population-based Cancer Registry (INPCR). Incidence rates were calculated using the population data available from the Statistical center of Iran. We considered the World standard population for calculation of age-standardized incidence rates (ASR). We also calculated 95% confidence intervals (CIs) for ASR. All rates are presented per 100000 person-years.

**Results:**

Overall, 19,679 new cases of EOGIC were registered by the INPCR between 2014 and 2018. The ASRs (95% CI) of EOEC, EOGC and EOCRC were 0.49 (95% CI: 0.47–0.51), 1.67 (1.63–1.71), and 3.07 (3.01–3.13) per 100,000 person-years, respectively. Our findings indicate decreasing and constant trends in the ASR of EOEC and EOGC during the study period, 2014-2018. There was an increasing trend in the ASR of EOCRC. We also found geographical disparities in the incidence rates of EOGICs across provinces of Iran, suggesting the highest ASRs of EOEC in Golestan (1.3), EOGC in Ilam (2.99) and EOCRC in Ilam (4.49).

**Conclusion:**

Our findings suggested that the incidence rate of EOCRC is consistently increasing. We also found variations in the incidence of EOGICs among different provinces. Further investigations are recommended to clarify the time trends and risk factors of EOGICs in Iran.

## Introduction

 In 2018, the World Health Organization reported that there were about 4.8 million fresh cases of gastrointestinal (GI) cancers globally, which resulted in 3.4 million related deaths. GI cancers account for 26% of the worldwide cancer incidence and 35% of all cancer-caused fatalities. Esophageal, gastric, and liver cancers were more prevalent in Asia, while colorectal and pancreatic cancers were more prevalent in Europe and North America.^1,2^ As one of the most common malignant tumors, GI cancer poses a serious threat to human health. GI cancer frequently occurs in middle-aged and elderly people, with the highest incidence in people aged 50 to 70.^3^ However, In spite of overall decreasing GI cancer rates, particularly in patients older than 50 years,^4^ the trend is the opposite in younger patients.^5,6^ In the United States, the age-adjusted incidence of early-onset colorectal cancer rose from 7.9 to 12.9 cases per 100 000 people between 1988 and 2015, a 63% increase.^7^

 Early-onset gastrointestinal cancers (EOGIC) has different definitions in studies, such that Strong et al. have introduced EOGIC in patients under 40 years of age.^8^ Still, the most common definition for EOGIC includes patients under 50 years of age with various cancers of the GI tract with a considerable mortality rate.^9^ Based on a study by Patel and Ahnen in the United States, colorectal cancer is the third most common cause of cancer mortality among people under 50 years old.^10^ The incidence of EOGIC has been on the rise over the past four decades^10^ and is expected to increase by > 140% by 2030.^6,11^ Early-onset colorectal cancer (EOCRC) is more prevalent in Asia and the Middle East, with 20% of cases compared to 2-8% reported in the United States.^12,13^ The incidence of EOGIC is increasing in several countries, including the United States,^14^ France,^15^ Canada,^16^ Australia,^17^ Japan, South Korea, Taiwan,^18^ Iran^19^ and Oman.^20^ CRC is the top cancer among men and second in women in Oman. Incidence has risen in the past 20 years,^21^ with 30% of cases diagnosed in patients younger than 50.^20^ In Iran, the incidence of cancer was 19.4 and 17.2 per 100 000 in males and females, respectively. The three most common GI cancers in males (by age-standardized rate) were esophageal cancer (4.7 per 100 000), stomach cancer (3.7 per 100 000), and colorectal cancer (2.9 per 100 000). In females, the three most common GI cancers were colorectal cancer (4.3 per 100 000), stomach cancer (2.5 per 100 000), and esophageal cancer (2.03 per 100 000).^22^

 The reasons for the rising incidence, mortality, and risk factors of EOGIC are unclear. However, increasing adiposity, unhealthy behaviors, and the global westernization of diets are considered risk factors.^23^ Stress, antibiotics, synthetic food dyes, physical inactivity, and sedentary behavior may increase the risk of EOGIC, which is also influenced by gut microbiota.^13,23,24,25^ Genetic and non-genetic risk factors, as well as stages of EOGI disease development, have also been reported. Some single nucleotide polymorphisms (SNPs) have been associated with a higher risk of gastric cancer. In contrast, combined analysis of multiple SNPs may lead to a high-risk classification for a specific population.^26^

 However, the results of these studies discuss the inconsistent characteristics and unclear risk factors of EOGIC. Identifying these factors is important due to the high mortality rate of EOGI patients. as well as in GLOBOCAN 2018 mentioned the potential doubling of colorectal cancer incidence in Iran before 2040.^27^

 Recent reports from Iran suggested increasing trends in incidence rates of CRC and geographical disparities in the incidence rates of GI cancers across Iranian provinces.^28,29,30,31,32^ Regarding limited information on EOGICs in Iran, we aim to present incidence rates and geographical distribution of most common EOGICs in Iran, 2014-2018.

## Materials and Methods

 This cross-sectional study was undertaken to investigate the incidence rates of the most EOGIC in Iran between 2014-2018. The data on new cases of the most common EOGICs were extracted from the Iranian National Population-based Cancer Registry (INPCR) annual reports, which are publicly available and published by the Iranian Ministry of Health.^33-37^ The INPCR has previously published details of the standard protocols and instructions utilized for the registry.^28,29^

 For this study, data on early-onset (age below 50 years) common GI cancers, including esophageal, gastric, and colorectal cancers, were extracted from the INPCR annual reports.^33-37^ The number and incidence rates of early-onset esophageal cancer (EOEC), early-onset gastric cancer (EOGC), EOCRC were calculated by gender, diagnosis year, and province. The truncated age-standardized incidence rates (ASRs) (0-49 years) were calculated using the World standard population and direct standardization method.^38^ All incidence rates were presented in 100 000 person-years. The analysis was carried out using the R program.

## Results

 The INPCR reported a total of 19 679 new cases of EOGIC between 2014 and 2018. The registered cases included 1798 cases of EOEC, 6251 EOGC, and 11,630 cases of EOCRC. The quality of data was assessed using the percentage of microscopic verification (MV%), which was 82.6% (n = 1486), 79.7% (n = 4984), and 88.0% (n = 10 232) for EOEC, EOGC, and EOCRC, respectively.

 The ASRs for Iranian population were 0.49 (95% confidence interval [CI]: 0.47–0.51), 1.67 (1.63–1.71), and 3.07 (3.01–3.13) per 100 000 person-years for EOEC, EOGC, and EOCRC, respectively. Table 1 presents the crude rate, ASR, and 95% CI of ASR for EOGICs in Iran between 2014 and 2018. Furthermore, the ASRs of EOEC were 0.51 (male) and 0.46 (female) per 100 000 person-years, respectively. The incidence rate of EOGC was higher in males (ASR = 1.90) than females (1.43). The ASRs of EOCRC were 3.13 and 3.01 in males and females, respectively.

**Table 1:** The number, crude rate, age standardized incidence rate (ASR) (per 100 000 person-years) and 95% confidence interval (CI) of ASR for early-onset esophageal cancer (EOEC), early-onset gastric cancer (EOGC) and early-onset colorectal cancer (EOCRC) in Iran, 2014-2018

**Cancer type**	**Population**	**Number**	**Crude**	**ASR**	**95% CI of ASR**
EOEC	Both male and female	1798	0.55	0.49	0.47-0.51
	Male	961	0.58	0.51	0.47-0.55
	Female	837	0.52	0.46	0.42-0.5
EOGC	Both male and female	6251	1.92	1.67	1.63-1.71
	Male	3553	2.15	1.9	1.84-1.96
	Female	2698	1.69	1.43	1.37-1.49
EOCRC	Both male and female	11630	3.58	3.07	3.01-3.13
	Male	6021	3.64	3.13	3.05-3.21
	Female	5609	3.51	3.01	2.93-3.09

**Figure 1:**
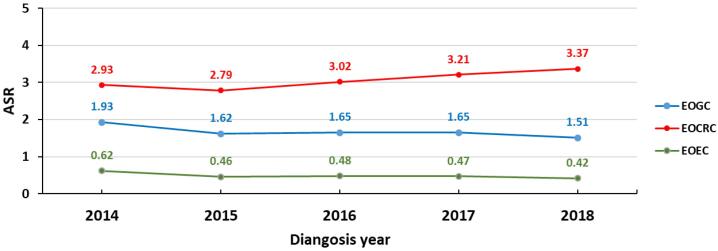
Time trends of the age standardized incidence rates (ASR) (per 100 000 person-years) of early-onset esophageal cancer (EOEC), early-onset gastric cancer (EOGC) and early-onset colorectal cancer (EOCRC) in Iran, 2014-2018

**Figure 2:**
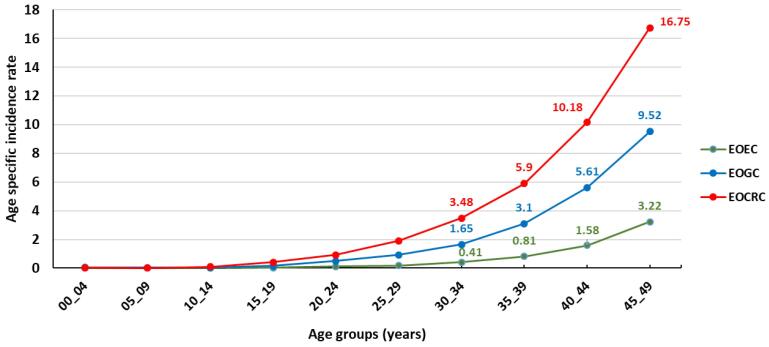
Age specific incidence rates (per 100 000 person-years) of early-onset esophageal cancer (EOEC), early-onset gastric cancer (EOGC) and early-onset colorectal cancer (EOCRC) in Iran, 2014-2018

Table 2 shows the number and incidence rates of EOGICs by diagnosis year, while Figure 1 illustrates the time trends in the ASR of EOEC, EOGC, and EOCRC in the Iranian population during the study period. The findings indicate a decreasing trend in the ASR of EOEC. The incidence rates of EOGC remained relatively stable during the study period, while there was an increasing trend in the ASR of EOCRC. Figure 2 presents the age-specific incidence rates (per 100 000 person-years) of EOGICs in Iran between 2014 and 2018, which suggest higher rates of EOCRC in young adults in Iran.

**Table 2:** The number, crude rate, age standardized incidence rate (ASR) (per 100 000 person-years) and 95% confidence interval (CI) of ASR for early-onset esophageal cancer (EOEC), early-onset gastric cancer (EOGC) and early-onset colorectal cancer (EOCRC) in Iran by diagnosis year, 2014-2018

**Cancer type**	**Diagnosis year**	**Number**	**Crude**	**ASR**	**95% CI**
EOEC	2014	425	0.67	0.62	0.56-0.68
	2015	335	0.52	0.46	0.4-0.52
	2016	360	0.55	0.48	0.42-0.54
	2017	351	0.53	0.47	0.41-0.53
	2018	327	0.49	0.42	0.38-0.46
EOGC	2014	1354	2.14	1.93	1.83-2.03
	2015	1183	1.83	1.62	1.52-1.72
	2016	1235	1.89	1.65	1.55-1.75
	2017	1279	1.95	1.65	1.55-1.75
	2018	1200	1.81	1.51	1.43-1.59
EOCRC	2014	2081	3.29	2.93	2.79-3.07
	2015	2067	3.19	2.79	2.67-2.91
	2016	2297	3.52	3.02	2.9-3.14
	2017	2487	3.78	3.21	3.07-3.35
	2018	2698	4.07	3.37	3.23-3.51

Table 3 presents the number and incidence rates of EOGICs in Iran by provinces, indicating geographical disparities in the incidence rates of EOGICs across provinces of Iran. The highest ASR of EOEC was found in Golestan (ASR 1.3), Sistan-and-Baloochestan (ASR 1.29), and Ardabil (ASR 1.05) provinces. The incidence rates of EOGC were higher in Ilam (ASR 2.99), Ardabil (ASR 2.29), and West Azarbayjan (ASR 2.21) provinces. Similarly, the provinces with the highest rates of EOCRC include Ilam (ASR 4.49), Tehran (ASR 3.71), and Qom (ASR 3.68).

**Table 3:** The number, crude rate, age standardized incidence rate (ASR) (per 100 000 person-year) and 95% confidence interval (CI) of ASR for early-onset esophageal cancer (EOEC), early-onset gastric cancer (EOGC) and early-onset colorectal cancer (EOCRC) in Iran by provinces, 2014-2018

**Province**	**EOEC**				**EOGC**				**EOCRC**			
	**N**	**Crude**	**ASR**	**95% CI**	**N**	**Crude**	**ASR**	**95% CI**	**N**	**Crude**	**ASR**	**95% CI**
Alborz	52	0.47	0.38	0.28 - 0.48	192	1.74	1.36	1.16 - 1.56	420	3.81	3.01	2.72 - 3.3
Ardabil	63	1.21	1.05	0.8 - 1.3	139	2.68	2.29	1.9 - 2.68	199	3.83	3.23	2.78 - 3.68
Busher	10	0.2	0.18	0.06 - 0.3	49	0.98	0.84	0.59 - 1.09	121	2.41	2.35	1.92 - 2.78
Chaharmahal and Bakhtiari	30	0.76	0.77	0.5 - 1.04	78	1.98	1.85	1.44 - 2.26	99	2.51	2.31	1.84 - 2.78
East Azarbayjan	146	0.94	0.78	0.64 - 0.92	384	2.47	2.05	1.83 - 2.27	632	4.07	3.3	3.05 - 3.55
Fars	40	0.2	0.19	0.13 - 0.25	384	1.95	1.67	1.49 - 1.85	613	3.11	2.68	2.46 - 2.9
Ghazvin	21	0.4	0.35	0.19 - 0.51	107	2.04	1.72	1.39 - 2.05	186	3.55	2.96	2.53 - 3.39
Golestan	107	1.37	1.3	1.05 - 1.55	180	2.31	2.09	1.78 - 2.4	308	3.95	3.62	3.21 - 4.03
Guilan	52	0.55	0.4	0.28 - 0.52	293	3.11	2.19	1.94 - 2.44	429	4.55	3.23	2.92 - 3.54
Hamedan	24	0.35	0.28	0.16 - 0.4	100	1.44	1.18	0.94 - 1.42	197	2.84	2.3	1.97 - 2.63
Hormozgan	25	0.32	0.37	0.21 - 0.53	113	1.45	1.52	1.23 - 1.81	174	2.24	2.26	1.91 - 2.61
Ilam	18	0.74	0.65	0.36 - 0.94	87	3.58	2.99	2.34 - 3.64	128	5.27	4.49	3.69 - 5.29
Isfahan	58	0.29	0.23	0.17 - 0.29	389	1.92	1.54	1.38 - 1.7	826	4.07	3.2	2.98 - 3.42
Kerman	44	0.33	0.34	0.24 - 0.44	259	1.94	1.87	1.63 - 2.11	264	1.98	1.87	1.63 - 2.11
Kermanshah	32	0.41	0.34	0.22 - 0.46	115	1.47	1.2	0.98 - 1.42	264	3.37	2.73	2.4 - 3.06
Khoozestan	81	0.4	0.4	0.3 - 0.5	304	1.51	1.43	1.27 - 1.59	637	3.17	2.99	2.75 - 3.23
Kohkilooye and Boyerahmad	16	0.52	0.53	0.26 - 0.8	55	1.8	1.83	1.34 - 2.32	77	2.52	2.47	1.9 - 3.04
Kordestan	43	0.65	0.58	0.4 - 0.76	105	1.59	1.42	1.15 - 1.69	185	2.8	2.37	2.02 - 2.72
Lorestan	30	0.41	0.37	0.23 - 0.51	147	2.02	1.84	1.55 - 2.13	194	2.66	2.29	1.96 - 2.62
Markazi	22	0.39	0.31	0.17 - 0.45	82	1.45	1.17	0.92 - 1.42	201	3.54	2.96	2.55 - 3.37
Mazandaran	71	0.56	0.43	0.33 - 0.53	366	2.88	2.15	1.93 - 2.37	586	4.61	3.53	3.24 - 3.82
North Khorasan	34	0.95	0.89	0.58 - 1.2	48	1.34	1.33	0.96 - 1.7	87	2.43	2.35	1.84 - 2.86
Qom	20	0.46	0.45	0.25 - 0.65	97	2.21	2.03	1.62 - 2.44	182	4.15	3.68	3.13 - 4.23
Razavi Khorasan	241	0.9	0.85	0.73 - 0.97	508	1.9	1.78	1.62 - 1.94	972	3.64	3.35	3.13 - 3.57
Semnan	11	0.39	0.34	0.14 - 0.54	42	1.48	1.29	0.9 - 1.68	108	3.81	3.37	2.72 - 4.02
Sistan and Baloochestan	124	0.99	1.29	1.05 - 1.53	170	1.36	1.64	1.39 - 1.89	174	1.39	1.72	1.45 - 1.99
South Khorasan	19	0.6	0.6	0.33 - 0.87	43	1.36	1.4	0.97 - 1.83	90	2.84	2.81	2.22 - 3.4
Tehran	204	0.39	0.31	0.27 - 0.35	940	1.79	1.39	1.29 - 1.49	2524	4.81	3.71	3.55 - 3.87
West Azarbayjan	111	0.82	0.75	0.61 - 0.89	328	2.43	2.21	1.97 - 2.45	453	3.36	2.97	2.7 - 3.24
Yazd	27	0.57	0.58	0.36 - 0.8	70	1.48	1.38	1.05 - 1.71	183	3.88	3.61	3.08 - 4.14
Zanjan	22	0.51	0.47	0.27 - 0.67	77	1.78	1.62	1.25 - 1.99	117	2.71	2.34	1.91 - 2.77

## Discussion

 To our knowledge, this is the largest descriptive study to describe the epidemiology of EOGICs in Iran. We provided an overview of data of early-onset (age below 50 years) common GI cancers, including esophageal cancer, gastric cancer, and colorectal cancer were extracted from the annual reports of the INPCR.

 We found that the incidence of EOCRC increased continuously over a long period of time, while the incidence of EOGC remained stable, and the ASR of EOEC decreased. These trends were observed not only at the national level but also at the regional level. For instance, a study conducted in Italy revealed that the incidence rate of colorectal cancer among individuals aged between 20 and 49 years increased from 9.3 in 1957 to 13.7 in 2015.^4^ Meanwhile, another study conducted in the United States by Glover et al. reported that the overall incidence rate of EOCRC was 18.9 per 100 000, and this type of cancer was more common among Caucasian and female populations.^12^ Additionally, a cohort study conducted in Canada showed that the ASR of EOCRC increased by 6.7% per year for 15-29 year-olds, by 2.4% per year for 30-39 year-olds, and by 0.8% per year for 40-49 year-olds between 1997 and 2010.^16^ Sung et al. conducted a study in Taiwan and found that the incidence of EOCRC significantly increased in both men and women, while in Korea, the incidence of EOCRC significantly increased in both genders. However, in Japan and Hong Kong, only a significant increase in rectal cancer was noted.^18^ On the other hand, Hang et al. reported that EOEC decreased in the United States from 2010 to 2017, with an annual percent change of -5.78%.^39^ Finally, a study conducted in China reported that the standardized incidence of EOGC decreased from 5.49/100 000 in 2000 to 4.76/100 000 in 2019.^40^ We conducted a study to determine the incidence rates of EOEC, EOGC, and EOCRC in the Iranian population using age standardization. According to our findings, the ASRs per 100 000 person-years for EOEC, EOGC, and EOCRC were 0.49 (95% CI: 0.47-0.51), 1.67 (95% CI: 1.63-1.71), and 3.07 (95% CI: 3.01-3.13), respectively. Males had higher ASR rates than females for EOEC (0.51 for males and 0.46 for females), EOGC (1.90 for males and 1.43 for females), and EOCRC (3.13 for males and 3.01 for females) per 100 000 person-years.

 The data between 2014 and 2018 shows that the incidence rate of EOCRC is increasing (Figure 1). The analysis of the data based on ASR indicates that EOCRC has a higher incidence rate than EOGC and EOEC cancers in all age groups. The difference in incidence increases with age, with the ASR rate of cancers at ages 30-34 being 3.48 for EOCRC, 1.65 for EOGC, and 0.41 for EOEC. At ages 45-49, the ASR rate of cancers is 16.75 for EOCRC, 9.52 for EOGC, and 3.42 for EOEC (Figure 2). Therefore, it is crucial to pay attention to this increase in the incidence of EOCRC and take preventive measures.

 In the survey conducted between the provinces of Iran, the results indicated that there are geographical differences in the incidence of EOGIC. EOEC incidence in Golestan (ASR 1.3) was the highest, followed by Sistan and Baloochestan (ASR 1.29), Ardabil (ASR 1.05), and North Khorasan (ASR 0.89) provinces. The EOGC incidence was highest in Ilam (ASR 2.99), followed by Ardabil (ASR 2.29), West Azarbayjan (ASR 2.21), and Guilan (ASR 2.19) provinces. However, during the study period, it was observed that the incidence of EOCRC increased significantly and had a higher incidence rate compared to EOEC and EOGC. The highest rates of EOCRC were observed in the provinces of Ilam (ASR 4.49), Tehran (ASR 3.71), Qom (ASR 3.68), and Yazd (ASR 3.61).

 As the main limitation of the present study, due to availability of short-term data (only 5 years), we could not perform robust analysis on time trends of EOGICs. Therefore, further investigations are recommended to assess temporal trends of EOGICs in Iran, upon availability of long-term data.

## Conclusion

 Our study offers a comprehensive overview of the epidemiology of early-onset GI cancers in Iran, and we have observed that the incidence rate of early-onset colorectal cancer is consistently increasing. However, the incidence rates of early-onset gastric and esophageal cancers are either decreasing or remaining stable, which is in line with findings from other countries. Our research has also revealed that there are variations in the incidence of early-onset GI cancers among different provinces in Iran. In view of the increasing incidence of early-onset colorectal cancer, it is essential to implement preventive measures to address this issue. All in all, our study emphasizes the significance of continuous monitoring and surveillance of early-onset GI cancers to inform public health interventions and improve outcomes for patients.
